# Biometric Indicators, Gelatin Yield, and Functional Properties of Fish Scale Waste From Three Species With Different Trophic Levels

**DOI:** 10.1155/ijfo/6628788

**Published:** 2025-06-23

**Authors:** Hafrijal Syandri, Netti Aryani, Azrita Azrita

**Affiliations:** ^1^Departement of Aquaculture, Faculty of Fisheries and Marine Science, Bung Hatta University, Padang, Indonesia; ^2^Departement of Aquaculture, Faculty of Fisheries and Marine Science, Riau University, Pekanbaru, Indonesia

**Keywords:** biochemical composition, biometric analysis, fish scale waste utilization, gelatin extraction, trophic level influence

## Abstract

Gelatin is a widely used food additive due to its ability to form gels and provide elastic texture to various food and feed products. This study evaluated the biometric characteristics as well as the physicochemical and functional properties of gelatin derived from waste scales of *Puntioplites bulu* fish (herbivore), *Cyprinus carpio* (omnivore), and *Channa lucius* (carnivore). The scales were first treated with 3% (*v*/*v*) glacial acetic acid and extracted with distilled water (1:4, *w*/*v*) at 80°C for 4 h. Fulton's condition factor ranged from 1.13 to 3.30. The length–weight relationship showed positive (+) and negative (−) allometric growth patterns. The average gelatin yield (based on wet weight) was 12.35% (*P. bulu*), 7.13% (*C. carpio*), and 5.03% (*C. lucius*). The extracted gelatin had an acidic pH (6.09–6.28). The foaming ability (FA) and foaming stability (FS) values were *P. bulu* (613.33% and 83.42%), *C. carpio* (416.67% and 28.3%), and *C. lucius* (550% and 73.06%). Furthermore, the water holding capacity (WHC) and fat binding capacity (FBC) of fish scale gelatin were 777.33% and 98.33% (*P. bulu*), 312% and 37.66% (*C. carpio*), and 967.0% and 207.33% (*C. lucius*), respectively. The gel strength and viscosity of *P. bulu* (226.67 g and 4.83 cP), *C. carpio* (207.33 g and 5.37 cP), and *C. lucius* (278.33 g and 6.90 cP) were measured. In conclusion, gelatin from fish scale waste at different trophic levels exhibits varied biometric, physicochemical, and functional properties, with potential applications in human and animal food industries.

## 1. Introduction

Aquaculture is renowned for its contribution to the global demand for fishery products driven by economic and demographic growth [1]. It contributes to over half of the world's fish supply and eases the strain on wild fish stocks [2]. Large amounts of nutrients are produced during the industrial manufacturing of fishery products with added value [3]. However, it also generates large amounts of waste [4, 5], including fish scales [6–8].

Biorefinery-based methods using biotechnology have significant potential to process fish industry waste on a commercial scale [9]. Due to the functional properties, isolated fish waste products can be useful in health and food to support Sustainable Development Goals (SDGs) achievement, specifically food security and nutrition, as well as environmental sustainability [10, 11].

The head (21.5%), viscera (17%), skin (3.3%), backbone (15.3%), fins (6.1%), and scale (8%) constitute over 71.2% of the total fish weight that is abandoned during the processing into fillets in the catch fisheries and aquaculture business [5, 12]. Although scale only makes up a small portion of the total weight, this part has the potential to be processed into flour [13], as well as products including gelatin and collagen, which are used in the cosmetic, pharmaceutical, and food industries [14, 15].

Collagen from fish scale, bones, and skin is partially hydrolyzed to generate the protein known as gelatin [12]. Collagen, the primary protein, becomes denatured when heated to create gelatin [16], which is among the most commonly used food additives due to its ability to form gels and provide elastic texture to various food products. The easily digestible nature makes gelatin an ideal component in certain foods, specifically those that require high protein content and are easily absorbed [17]. However, the use of powdered gelatin obtained from fish scale as an ingredient in fish fry feed remains underexplored, particularly in efforts to support the diversification of food and feed resources.

Fish gelatin is a hydrolyzed collagen product widely utilized as a versatile natural biomaterial in various industries, including food, pharmaceuticals, biomedicine, and the cosmetic industry [12, 14, 18]. Several studies have examined the characteristics and potential of gelatin derived from fish waste [19, 20], including fish scales, as an alternative source of nonmammalian gelatin [21]. The advantages of fish gelatin lie in its physicochemical and functional properties, such as solubility, gel strength, viscosity, gelling ability, and stability under specific temperature and pH conditions [22, 23]. Studies on gelatin extracted from fish skin, bones, and scales have shown that its composition and quality are influenced by various factors, including fish species [24, 25], extraction methods, and pretreatment processes [23, 26]. The application of hydrolysis methods, whether acid, alkaline, or enzymatic, has been proven to affect the yield, mechanical properties, and rheological characteristics of the resulting gelatin [27].

Several freshwater fish species, such as *Oreochromis niloticus*, *Clarias batrachus*, *Pangasius sutchi* [24], *Channa micropeltes*, and *Channa striata* [28], as well as *Nemipterus japonicus* [29] and *Labeo rohita* [30], have been observed to extract gelatin from their skins and bones. However, studies on gelatin extraction from fish scale waste and the characterization of the functional properties based on trophic levels of herbivores, omnivores, and carnivores remain very limited.

We selected three fish species based on their trophic levels and economic importance. The three freshwater species—*Puntioplites bulu* (herbivore), *Cyprinus carpio* (omnivore), and *Channa lucius* (carnivore)—are widely traded in local markets and play a significant role in small-scale fisheries [31]. However, fish scale waste generated from processing these species remains underutilized, limiting its potential contribution to circular economy initiatives [32]. Given the differences in trophic levels and economic value, there is still a research gap in understanding how trophic factors influence fish biometric parameters as well as the physicochemical and functional properties of the extracted gelatin.

We hypothesized that biometric indicators and characteristics of gelatin obtained from three fish species at different trophic levels herbivorous—(*P. bulu*), omnivorous (*C. carpio*), and carnivorous (*C. lucius*)—show variations in condition factors (CFs), growth patterns, gelatin production, foaming ability (FA) and foaming stability (FS), water holding capacity (WHC), lipid binding capacity, gelatin strength, viscosity, and biochemical composition. Therefore, this study was aimed at evaluating the biometric data of the sampled species, gelatin production, functional properties, and nutritional composition as an alternative gelatin source, which not only increases the added value of fishery by-products but also contributes to waste reduction and sustainable industry development.

## 2. Material and Methods

### 2.1. Animal Materials

A total of 25 *P. bulu*, *C. carpio*, and *C. lucius* were provided by small-scale fishermen from Kampar Kanan River, Kampar Regency, Riau Province, Indonesia, which were gathered between July and September 2024. Each fish species comes from the same habitation, namely, the Kampar Kanan River area. However, in this study, the age and sex of the fish were not determined. Then, immediately after being placed in an ice box, the fresh fish were brought to the laboratory and verified by the Fisheries Department, Faculty of Fisheries and Marine Science, Bung Hatta University, Indonesia.

To ensure the accuracy of the equipment, the OHAUS scale (model CT 6000, United States), stainless steel caliper, digital pH meter, and other tools were calibrated before use. This calibration process was conducted to ensure precise measurements of fish weight, fish scales, gelatin weight, gelatin pH, and other parameters analyzed in the study.

Each fish was weighed separately (TW) in the lab using an OHAUS (model CT 6000 United States) balancing scale with a 0.1 g precision, and its maximum body height (H) and standard length (SL) were measured. The H was measured vertically, without the fins, and the SL was measured from the tip of the mouth to the top lobe of the caudal fin. A digital caliper with an accuracy of 0.01 mm was used to measure body H, and a SL was measured with a ruler that was precise to 1 mm. The following formula was used to calculate the Fulton CF [33]:

(1) $$\begin{array}{c} \text{CF}=\left(\frac{\text{TW}}{{\text{SL}}^{3}}\right)\times 100. \end{array}$$

The growth pattern was evaluated based on the value of *b* using the regression equation described in [34]:

(2) $$\begin{array}{c} W={aL}^{b}, \end{array}$$

where *W* is the body weight (grams), *L* is the total length (centimeters), *a* is the intercept (constant), and *b* is the regression coefficient representing the growth pattern.

### 2.2. Fish Scale Preparation

Fish scales from three selected freshwater species, caught by small-scale fishermen in Kampar Kanan River, were collected from vendors at traditional markets in Kampar area, Kampar Regency, Riau Province. A stainless-steel fish scaler measuring 17.5 cm by 3.5 cm by 1.5 cm was used to remove the scales. The size of each species' scale was measured using a digital caliper with an accuracy of 0.01 mm.

### 2.3. Pretreatment and Extraction of Gelatin From Fish Scale

Fish scales from each species were initially rinsed with water to remove surface debris and briefly air-dried. The scales were then immersed in a 3% (*v*/*v*) glacial acetic acid solution at a scale-to-solution ratio of 1:4 (*w*/*v*), corresponding to 200 g of scales soaked in a mixture of 24 mL glacial acetic acid and 776 mL distilled water. The soaking process was carried out in a 1000 mL graduated cylinder at room temperature for 48 h. After soaking, the scales were thoroughly washed several times with distilled water until the pH of the rinsing solution reached neutrality (pH 7). The use of 3% (*v*/*v*) glacial acetic acid was adapted from the method of Zuraida and Pamungkas [35], with slight modifications.

Following hydrolysis, the fish scales were rinsed four times with distilled water, each rinse lasting 15 min. Gelatin extraction was then carried out by immersing the scales in distilled water at a scales-to-solution ratio of 1:4 (*w*/*v*) and heating the mixture at 80°C for 4 h. The extraction temperature was adopted from the procedure described by [35]. Whatman Grade 1 filter paper measuring 110 mm was used to filter the extraction products.

After filtration, the filtrate was placed into a 20 × 15 cm plastic tray that was 0.3 cm thick; it was left to dry for 48 h at room temperature (28°C–30°C) until gelatin sheets formed. Subsequently, the gelatin sheets were weighed every 6 h until two consecutive weighings showed a weight difference of less than 0.01 g, which typically required 2–3 weighings. The drying process was considered complete once the gelatin sheets reached a constant weight. This approach was used to ensure that the gelatin sheets were sufficiently dried for subsequent analysis.

After drying, gelatin sheets were cut into 3 × 2 cm pieces and ground into powder using a Miller Powder Grinder (Miller Fomac FCT-Z100, Tiongkok). To create a fine gelatin powder, the powder was sieved using a 150 *μ*m sieve. Lyophilization was not performed in this process.

Additionally, the proximate composition, mineral content, and amino acid content of gelatin powder for each fish species were examined. Although the acid extraction method used in this study is effective in obtaining gelatin, there is a possibility of losing some functional properties during processing. Additionally, the extraction efficiency may be influenced by conditions such as pH, temperature, and reaction time, which require further optimization.

The following formula was used to determine the yield based on both weight and protein content [24]:

(3) $$\begin{array}{c} \left(\text{i}\right)\;\%\text{Yield }\left(\text{wet weight basis}\right)=\frac{\text{Dry weight of gelatin}}{\text{Wet weight of scale}}\times 100. \end{array}$$

(4) $$\begin{array}{c} \left(\text{ii}\right)\;\%\text{Yield }\left(\text{dry weight basis}\right)=\frac{\text{Dry weight of gelatin}}{\text{Wet weight of scales}-\text{moisture content}}\times 100. \end{array}$$

(5) $$\begin{array}{c} \left(\text{iii}\right)\;\%\text{Yield }\left(\text{protein basis}\right)=\frac{\text{Dry weight of gelatin}}{\text{Protein content of scales}}\times 100. \end{array}$$

### 2.4. pH

A 1.0 g sample of gelatin was dissolved in 100 mL of distilled water, and the pH was measured at 25°C [36].

### 2.5. Foam-Forming Ability and FS

FA was assessed based on the initial foam volume, while FS was determined by measuring the change in foam volume over time, following the method described by Cho et al. [37]. To allow for swelling, a gelatin solution (1 g/100 mL) was heated to 60°C. The initial sample volume was recorded as *Vo*. A magnetic stirrer was used to constantly homogenize the solution for 3 min to produce foam. The volume of foam without liquid was recorded as *V*. After 30 min, the volume of foam without liquid was noted as *V*_1_. The following formulas were then used to calculate the FA and FS:

(6) $$\begin{array}{c} \text{FA }\left(\%\right)=\left(\frac{V}{Vo}\right)\times 100. \end{array}$$

(7) $$\begin{array}{c} \text{FS}\left(\%\right)=\left(\frac{V_{1}}{V}\right)\times 100. \end{array}$$

### 2.6. WHC and Fat Binding Capacity (FBC)

With minor adjustments, the technique of Cho et al. [37] was used to evaluate the WHC and FBC of gelatin. In brief, 1.0 g of gelatin was placed in a graduated cylinder and mixed with 10 mL of distilled water (for WHC) or maize oil (for FBC). The mixture was then allowed to stand at room temperature for half an hour. Afterward, the excess water or maize oil was removed by filtration, and the moist gelatin was weighed. The WHC and FBC values were calculated using the following formula:

(8) $$\begin{array}{c} \left.\frac{\text{WHC}}{\text{FBC}}\left(\%\right)\right)=\left(\frac{W1-W0}{W0}\times 100\%\right), \end{array}$$

where *W*_1_ is the weight of the sample after water/fat absorption (in grams) and *W*_0_ is the initial dry weight of the sample (in grams).

### 2.7. Gel Strength

Gelatin gel strength was evaluated following the procedure described by Liao et al. [38], with slight modifications by Asiamah et al. [39]. 7.5 g of gelatin was dissolved in a 150 mL bloom bottle containing 105 mL of distilled water to obtain a 6.67% solution. The solution was allowed to hydrate at room temperature for approximately 3 h, then heated to 40°C for 30 min using a magnetic stirrer. After heating, the solution was cooled for 15 min before being stored at 4°C (±1°C) for 17 h (±1 h) prior to analysis. Gel strength was determined using a TAXT2 texture analyzer (Stable Micro System, United Kingdom) equipped with a 0.5-in. diameter cylindrical probe. The probe was lowered at a speed of 0.5 mm/s to a penetration depth of 4 mm, with the maximum force recorded during penetration representing the Bloom strength. The results were expressed as mean values ± standard deviation (*SD*) from three replicates.

### 2.8. Viscosity

The viscosity of gelatin was measured using an Ostwald viscometer (Type No. 4), which is designed for high-viscosity liquids. A solution was prepared by dissolving 6.67 g of gelatin in 100 mL of distilled water, followed by heating at 60°C until completely dissolved. The viscosity was then assessed at room temperature following the method described by Shyni et al. [40]. The results were expressed as mean values ± *SD* from three replicates.

### 2.9. Nutritional Evaluation of Gelatin Powder

Gelatin samples (in the form of powder) were analyzed for proximate composition using AOAC standard techniques [41]. Protein content in the three samples was analyzed using the Kjeldahl method (AOAC 984.13), applying a nitrogen-to-protein conversion factor of 5.55, as recommended for gelatin [42]. Fat content was analyzed using the Soxhlet method (AOAC 920.39), ash content through incineration (AOAC 942.05), and moisture content using the gravimetric method (AOAC 930.15).

The purpose of the analysis using high-performance liquid chromatography (HPLC) in this study is to identify and quantify the amino acid content in gelatin derived from fish scales. The procedures described by Cohen [43] were followed for performing amino acid analysis. HPLC, a Waters 1525 binary pump, a Waters 717 autosampler (Waters), and a Waters 2475 multi-*λ* fluorescence detector with wavelengths set at 250 nm for excitation and 395 nm for emission, was used to assess the amino acid profile. For 24 h at 110°C, the materials were hydrolyzed in triplicate using 6 N hydrochloric acid.

The ashed sample was dissolved in 1 mL of 35% *v*/*v* hydrochloric acid (Suprapur Merck) for the analysis of mineral composition, including Na, Mg, Ca, K, P, Zn, Fe, and Mn. The resulting solution was then diluted according to the specific requirements for each mineral analysis and filtered using cellulose filter paper (Whatman No.1, International Ltd., Maidstone, United Kingdom) to remove insoluble particles. Mineral content analysis was performed based on AOAC 985.35 using atomic absorption spectroscopy (AAS). A Perkin-Elmer AA Model 3110 (Norwalk, CT, United States) instrument was specifically used to determine the phosphorus (P) concentration in the sample.

### 2.10. Data Analysis

For each fish species, the analyzed parameters included gelatin yield, pH, FA, FS, WHC, FBC, gel strength, and viscosity. All parameters were subjected to a one-way analysis of variance (ANOVA) at a significance level of 0.05. When significant differences were detected, Duncan's multiple range test [44] was applied for post hoc comparisons.

To account for data variability, the distribution of each parameter was assessed using the Shapiro–Wilk test. All measurements were performed in triplicate, and results are reported as means ± SDs. However, amino acid and mineral analyses of the gelatin were conducted only once, without replication.

## 3. Results and Discussion

### 3.1. Biometric Indicators

The average wet weight, SL, H, CF, and scale size of the fish used as raw materials in this investigation to produce gelatin are shown in Table 1. Three chosen freshwater fish species had CFs greater than 1, which denotes high health and is a reflection of ideal feed quality and a supportive environment [11]. Fish at low trophic levels, such as *P. bulu* (herbivores), are more efficient in converting food into biomass than predators at high trophic levels. This efficiency makes herbivorous fish a sustainable resource to support aquatic ecosystems in line with SDGs 15. The *b* values of the three fish species revealed two growth patterns. *C. carpio* (2.6838) and *C. lucius* (2.7490) had *b* values less than 3, indicating negative allometric growth (body weight increases more slowly than body length). In contrast, *P. bulu* exhibited a *b* value of 3.3206, reflecting positive allometric growth (body weight increases faster than body length). Overall, a *b* value less than 3 indicates negative allometric growth, whereas a *b* value greater than 3 indicates positive allometric growth [45]. Growth patterns in fish species vary based on factors including fishing season, sampling location, species, specimen size, developmental stage, and trophic level [45, 46]. The *R*^2^ values were between 0.77 and 0.98, suggesting the accuracy and consistency of length–weight relationship (LWR) measurements, with each species showing a high significant correlation (*p* < 0.001) between the increase in length and body weight. The results are consistent with [47] who examined the same fish species in Koto Panjang Reservoir, Riau Province, Indonesia.

In addition, growth patterns may influence collagen content or scale structure, ultimately affecting gelatin extraction efficiency. In fish exhibiting negative allometric growth, such as *C. carpio* and *C. lucius*, body weight increases more slowly than body length, with average scale sizes of 15.04 and 10.54 mm, respectively. This condition may result in thinner or less dense scales, potentially leading to lower collagen concentrations or alterations in collagen organization, which could affect both the yield and quality of the extracted gelatin. Conversely, in species such as *P. bulu* that exhibit positive allometric growth, where body weight increases faster than body length, the scales, with an average size of 15.99 mm, may become thicker, denser, and richer in collagen, providing better raw material for gelatin production. Therefore, growth patterns not only reflect differences in somatic development but may also serve as an important biological factor influencing the characteristics and extractability of collagen from fish scales.

### 3.2. Yield of Gelatin

Table 2 presents the gelatin yield data based on wet weight, dry weight, and protein content. Significant differences (*p* < 0.05) in extraction yield were observed among the three freshwater fish species selected based on their trophic levels. The highest yield based on dry weight was obtained from the scales of the herbivorous species *P. bulu* (30.50% ± 2.34%), followed by the omnivorous *C. carpio* (20.98% ± 0.33%) and the carnivorous *C. lucius* (18.02% ± 0.20%). The superior yield from *P. bulu* scales may be attributed to their larger size, thicker structure, and higher collagen content compared to the other two species.

To address the variability of data, the distribution of each parameter was evaluated using the Shapiro–Wilk test, which revealed that most of the data were normally distributed (*p* > 0.05) and no extreme outliers were detected. In addition, the SDs across replicates were relatively low, indicating consistent results within each species. Minor variations were noted, particularly in the gelatin yield of *C. lucius*, which may be attributed to natural biological variability among individual fish or environmental influences.

The gelatin yields obtained in this study exceeded those reported by Jakhar et al. [30] for acid-extracted gelatin from the scales of *Catla catla*, *L. rohita*, and *Cirrhinus mrigala*, species that are predominantly omnivorous during their juvenile stages. However, the yields were slightly lower compared to other fish species reported in previous studies [24, 48]. Based on protein content, the gelatin yields from *C. carpio* and *C. lucius* were higher than those obtained from *Lutjanus campechanus* and *Epinephelus chlorostigma*, as reported by [49].

Previous studies have reported that gelatin yield can be influenced by the extraction method used [17, 23] and differences in fish species [25]. In addition, environmental factors such as season (dry and rainy) and fish habitat, whether in aquaculture or the wild, also contribute to variations in gelatin production in Nile tilapia. A study by Ref. [39] found that the scales of farmed Nile tilapia collected during the dry and rainy seasons produced higher gelatin yields (7.95% and 7.65%) compared to the scales of wild Nile tilapia collected in the same seasons (1.98% and 2.00%, respectively). In this study, three selected fish species were caught from the wild during the dry season between July and September 2024, with rainfall occurring between 6 and 9 days per month and total precipitation ranging from 113.3 to 130.1 mm [50]. Although this study does not specifically discuss the influence of environmental factors on gelatin properties, the fish samples were collected from the same habitat during the same season (dry season), ensuring that environmental factors such as water quality were assumed to be uniform. However, we acknowledge that environmental variability, including potential impacts of pollution or differences in natural habitats, may affect gelatin properties. Further studies are needed to evaluate how these factors contribute to the characteristics of the gelatin produced.

### 3.3. pH

pH values of gelatin from *P. bulu*, *C. carpio*, and *C. lucius* fish scale, using a 3% (*v*/*v*) acetic acid pretreatment method, were 6.09 ± 0.01, 6.16 ± 0.01, and 6.28 ± 0.05, respectively. Statistical analysis using ANOVA indicated a significant difference among the samples (*p* < 0.05). The levels showed minor variations across species. To address the variability of the data, the distribution of each parameter was evaluated using the Shapiro–Wilk test, which revealed that the data for each sample were normally distributed (*p* > 0.05) and no extreme outliers were detected. In comparison, Nile tilapia (*O. niloticus*) scale gelatin reportedly had a wider pH, ranging from 5.3–9.2 [40], similar to Type A and Type B gelatin from *L. rohita* scale, with pH values of 9.5 and 5.4, respectively [26]. The variation in pH values of fish scale gelatin is primarily influenced by differences in pretreatment methods used during extraction, which comprise both alkaline and acidic treatments [49].

### 3.4. FA and FS

**Figure 1:**
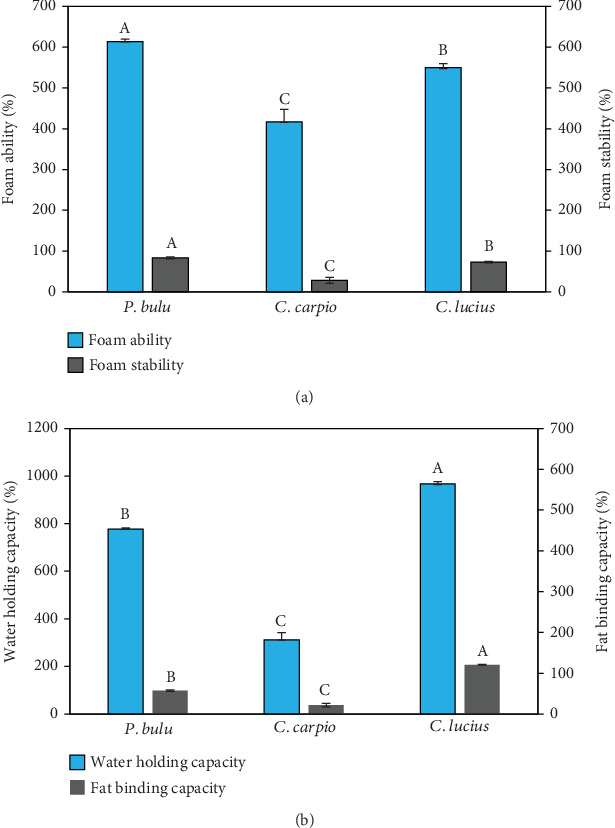
(a) The three samples' foam ability and foam stability. (b) The three samples' water holding capacity and fat binding capacity. Uppercase superscript letters A, B, and C denote significant variations among the three fish species (*p* < 0.05) *n* = 3.

In comparison to *C. lucius* (600% ± 10%) and *C. carpio* (406.67% ± 30.55%), the ratio of foam ability to the volume generated in fish scale gelatin from *P. bulu* (613% ± 5.77%) was substantially greater (*p* < 0.05). Conversely, the FS over the measurement period in *P. bulu* fish was significantly (*p* < 0.05) higher (83.42% ± 0.56%) than that of *C. lucius* (73.6% ± 1.76%) and *C. carpio* (28.30% ± 1.28%), as in presented Figure 1.

To assess the variability of the data, the distribution of each fatty acid and fish scale parameter was examined using the Shapiro–Wilk test. The results indicated that the data for most parameters followed a normal distribution (*p* > 0.05), with no significant outliers detected. Additionally, the SD across replicates was relatively small, suggesting a high degree of consistency in the results within each species.

In this study, the omnivorous fish *C. carpio* showed a lower ability to produce foam and a higher percentage of foam collapse. This is probably related to the mineral composition of gelatin, particularly the lower levels of magnesium, calcium, sodium, and potassium compared to *P. bulu* and *C. lucius*, which may have reduced its effectiveness in forming and stabilizing foam structure. Mineral ions such as calcium and magnesium are known to strengthen the interfacial film network around foam bubbles through cross-linking between protein molecules, thereby enhancing FS [15].

However, the differences in the foaming capacity of the gelatin could also be attributed to the content of hydrophobic amino acid residues [51]. In this study, the content of hydrophobic amino acids, such as leucine, valine, alanine, and glycine, was found to be lower in *C. carpio* compared to *P. bulu* and *C. striata*. This difference may contribute to the lower FA and higher foam collapse percentage in gelatin derived from *C. carpio*. Additionally, the total percentage of hydrophobic and hydrophilic amino acids was higher in *P. bulu* and *C. striata*, which may enhance the FA and FS of the gelatin obtained from these species.

### 3.5. WHC and FBC

As presented in Figure 1b, the WHC of fish scale gelatin varied significantly among species. The highest WHC was observed in *C. lucius*, reaching 967% ± 59.02%. This value was significantly higher (*p* < 0.05) compared to that of *P. bulu* (777.33% ± 24.46%) and *C. carpio* (312% ± 15.71%). The superior WHC in *C. lucius* can be attributed to its higher concentrations of hydrophilic amino acids such as lysine, arginine, histidine, and cysteine (Table 3). Moreover, the proline content in *C. lucius* was also slightly higher than in the other two species, which further contributed to its enhanced WHC. Previous studies have shown that WHC is positively correlated with the levels of hydroxyproline (Hyp) and proline [25].

To ensure the robustness of the WHC data, we performed a Shapiro–Wilk test, which indicated a normal distribution (*p* > 0.05) across all species. The data also exhibited relatively narrow SDs, suggesting good repeatability within replicates. Although *C. lucius* showed slightly greater variability than the others, this remains within acceptable limits and is likely associated with natural differences in biological composition or habitat-related factors.

The gelatin derived from *P. bulu* and *C. carpio* lacked cysteine, an amino acid known to enhance gelatin quality and functionality, particularly with respect to structural stability and water binding capacity (WBC). Similar findings have been reported for gelatin extracted from *E. chlorostigma* and *C. carpio* bones [49, 52]. Moreover, WHC was positively correlated with the levels of Hyp and proline [25], supporting the higher WHC values observed in *C. lucius*. The amino acid profiles underline the importance of protein composition in determining gelatin functionality.

FBC was significantly higher (*p* < 0.05) in *C. lucius* (207% ± 2.08%), compared to *P. bulu* (98.33% ± 2.51%) and *C. carpio* (37.66% ± 7.37%) (Figure 1b). The differences may be attributed to the distinct dietary patterns among the three freshwater fish species, which appear to influence both WBC and FBC of the produced gelatin. Notably, WHC and FBC followed consistent trends; for instance, *P. bulu* exhibited strong WHC but relatively low FBC. A high FBC is often associated with increased exposure to hydrophobic residues and elevated tyrosine content [52]. Although this study demonstrates that gelatin from *P. bulu* and *C. lucius* exhibits superior functional properties compared to *C. carpio*, its industrial-scale application remains limited. Further research is needed to assess large-scale production feasibility, cost efficiency, and comparisons with commercial gelatin to determine its industrial potential, including its use as an ingredient in aquaculture feeds, particularly for fish fry.

### 3.6. Gel Strength

Gel strength is an important parameter in assessing the functional properties of gelatin, as it reflects the average molecular weight of its components [38]. Gel strength also serves as a major criterion for classifying gelatin quality into three categories: low bloom (< 150), medium bloom (150–200), and high bloom (220–300). Previous studies have documented that the gel strength of fish gelatin ranges from 70 to 270 g/cm^3^, while mammalian gelatin ranges from 100 to 300 g/cm^3^ [53].

**Figure 2:**
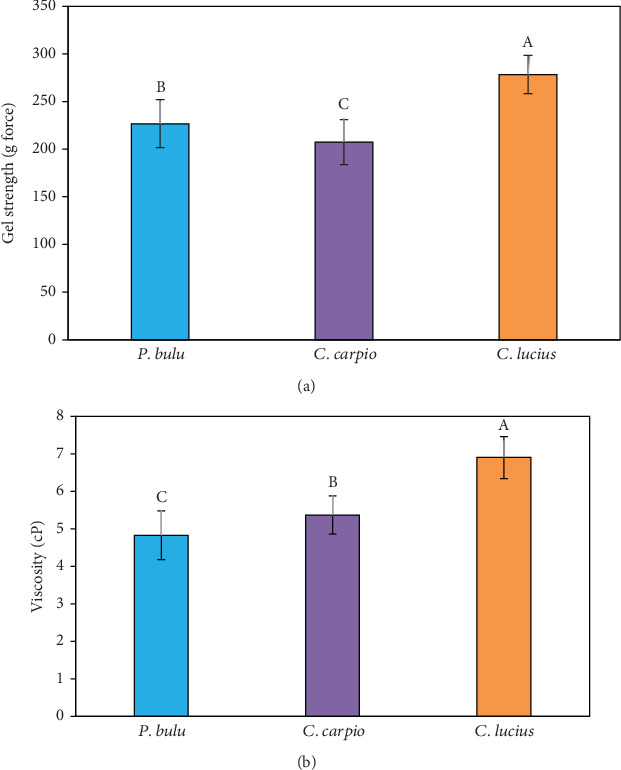
(a) Gel strength and (b) viscosity of gelatin extracted from three freshwater fish species. Uppercase superscript letters A, B, and C denote significant variations in three fish species (*p* < 0.05), *n* = 3.

In this study, the gel strength of gelatin extracted from the scales of *P. bulu* (226.67 ± 25.17 g), *C. carpio* (207.33 ± 23.59 g), and *C. lucius* (278.33 ± 20.21 g) showed statistically significant differences (*p* < 0.05) in gel-forming ability among these freshwater fish species (Figure 2a). To ensure data consistency, the Shapiro–Wilk test was performed and confirmed a normal distribution for all groups (*p* > 0.05), with no extreme outliers detected. The SDs were relatively moderate and consistent across species, suggesting acceptable reproducibility. Slight variations—particularly in *P. bulu*—may reflect natural biological variability or differences in the structural characteristics of collagen among species.

The observed variation in gel strength could be attributed to differences in the amino acid composition of gelatin from each species. Specifically, *C. lucius* gelatin contained 23.46% glycine, 11.01% proline, and 9.54% alanine, which were higher than the levels found in *P. bulu* (21.08% glycine, 10.72% proline, and 8.93% alanine) and *C. carpio* (21.05% glycine, 10.76% proline, and 8.75% alanine) (Table 3). According to He et al. [25], the gel strength of gelatin derived from fish scales was positively correlated with the content of proline and glycine. The amino acid composition of gelatin is similar to collagen, with Gly as the dominant amino acid. Gelatin is a linear polymer consisting of repeating Gly–Pro or Gly–Hyp units [22]. The findings of this study indicate that the gelatin obtained meets acceptable quality standards. In general, gelatin with lower gel strength produces a softer and less elastic texture, making it unsuitable for applications requiring a strong gel network [54].

In addition, He et al. [25] reported that the gel strength of gelatin extracted from carp (*C. carpio*) and bighead carp (*Hypophthalmichthys nobilis*) using alkali (NaOH) pretreatment reached 334.77 and 643.28 g, respectively. Meanwhile, gelatin derived from *C. striata* scales with alkali pretreatment showed a gel strength of 351.60 g [19]. These values are significantly higher than those obtained in the present study for *P. bulu*, *C. carpio*, and *C. lucius*, which used acid pretreatment.

Previous studies have also demonstrated that the gel strength of gelatin extracted from tilapia (*O. niloticus*) scales is affected by seasonal variations (dry and rainy seasons) and fish habitat (wild vs. farmed). Gelatin obtained from wild and farmed tilapia under these conditions showed gel strengths ranging from 100 to 120 g [39]. The impact of pretreatment, acid- and alkali-based extraction processes, and the influence of seasonal fluctuations and fish habitat need to be further examined. Given these variations, a comprehensive study is needed to evaluate how these factors affect the gel strength of gelatin obtained from the three fish species studied in this study.

### 3.7. Viscosity

Viscosity is an important functional characteristic for process control. The ability of a gelatin solution to form gel depends on its temperature and viscosity in water [55]. Several factors, including fish species, habitat, origin, and amino acid composition, affect the gel-forming quality of fish gelatin [24, 39]. Gelatin derived from warm water fish generally exhibits low viscosity, making it suitable for food industry applications [23].

In this study, the viscosity of gelatin obtained from the three fish species is presented in Figure 2b. Significant differences (*p* < 0.05) were observed, with *C. lucius* gelatin demonstrating the highest viscosity (6.90 ± 0.56 cP) followed by *C. carpio* (5.37 ± 0.51 cP) and *P. bulu* (4.83 ± 0.65 cP). The Shapiro–Wilk test confirmed that viscosity data were normally distributed (*p* > 0.05), with no outliers detected. The relatively low to moderate SDs across species indicate consistent and reproducible measurements. Slight variations, especially in *P. bulu*, are likely due to natural biological differences, such as variations in collagen molecular weight and cross-linking levels, which directly influence gelatin viscosity. The higher viscosity found in *C. lucius* may reflect the presence of higher molecular weight peptides and stronger intermolecular bonding, in line with prior studies linking viscosity with gelatin structure and amino acid content [39].

Comparative findings from other species further demonstrate the variability in gelatin viscosity, which is strongly influenced by fish species and tissue origin. For instance, gelatin extract from common carp scales recorded a viscosity of 26.3 cP [56], while lower values were reported for milkfish at 5.23 cP [57]. Conversely, slightly higher viscosities were found in lizardfish (7.5 cP) [58] and snakehead (10.25 cP) [19]. These variations underscore the role of species-specific biochemical characteristics in determining the functional properties of gelatin. Given these differences, further large-scale studies are warranted to evaluate the potential applications of fish scale–derived gelatin across various industries, including food, cosmetics, and fish fry feed formulations.

### 3.8. Proximate Chemical Composition of Fish Scale Gelatin

**Figure 3:**
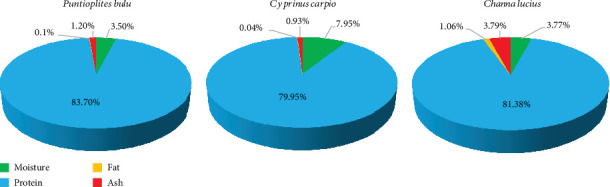
Biochemical composition of scale gelatin from three selected freshwater fish species.

Fish scale gelatin powder's moisture content varied from 3.77% to 7.95% (Figure 3), with lyophilization—rather than oven drying—being the cause of the low moisture content. At 3.20% and 3.58%, respectively, scale gelatin from wild and farmed Nile tilapia also showed reduced moisture levels [39]. According to reports, the average moisture content of fish scale gelatin was 8.36 ± 0.14 g/100 g for different species [12].

The protein level was 4% greater in *P. bulu* than in *C. carpio*, with the protein content ranging from 79.95% ± 0.01% to 83.70% ± 0.05%. The biochemical composition of scale gelatin from common carp (*C. carpio*) and big-headed carp (*H. nobilis*) also contains high protein, 92.45% and 92.55%, respectively. [25]. The protein content of gelatin recovered from fish scale has been reported in a number of investigations; the range of values is 58.45%–94.05% [59–62].

The scale gelatin also exhibited low fat contents (0.04%–0.10%) and ash contents (0.93%–3.79%), primarily due to the reduced mineral content present in the observation. For example, calcium and magnesium levels were 6.67 and 12.25 mg/kg, respectively, while the iron content was relatively low at 30.09% in *C. carpio* scale gelatin compared to *P. bulu* and *C. lucius* (Table 4). Lower ash contents in the scale gelatin of *O. niloticus* (1.19%–3.18%) were also reported [39], while another study examined the ash content of *H. nobilis* [62]. Ash content is closely associated with the mineral content [23]. Nonetheless, 2.6% is the highest amount of ash that is advised for gelatin [63].

### 3.9. Amino Acid Composition

Gelatin's chemical and physical characteristics are greatly influenced by its amino acid composition. The repeating sequence “Glycine-X-Y,” in which X is primarily proline and Y is often Hyp or sometimes alanine, primarily determines the structure [64]. In this study, glycine, alanine, and proline were the dominant amino acids in gelatin (Table 3). The high content of glycine is crucial in forming the spatial structure of gelatin [62]. Table 3 shows that gelatin from the scale of *P. bulu* and *C. carpio* contains low levels of tyrosine and methionine, and none contain cysteine. The outcomes are comparable to the amino acid composition of gelatin that was taken out of the carp scale, as shown by Ref. [25, 30]. In contrast, gelatin from the scale of Javanese fish (*C. lucius*) contains cystine, resembling the amino acid composition of snakehead fish (*C. striata*) [19, 65].

Based on the results, the amino acids were classified according to the properties of their side chains related to water solubility, distinguishing between hydrophobic and hydrophilic [52]. The hydrophobic and hydrophilic amino acid compositions of gelatin obtained from the fish scale of three species are shown in Table 3.

The total hydrophobic and hydrophilic amino acids for each species show interesting variations, which can influence the functional properties of the resulting gelatin, such as gel strength, texture, and its WBC. Gelatin extracted from the scales of *P. bulu* contains a total of 45.38% hydrophobic and 36.60% hydrophilic amino acids, while *C. carpio* has 42.65% hydrophobic and 37.38% hydrophilic amino acids. In contrast, *C. lucius* shows 46.68% hydrophobic and 31.69% hydrophilic amino acids.

These differences in composition reflect the distinct chemical characteristics between species, which may affect the gelatin's performance in industrial applications, including products that require high gel strength or good emulsifying properties. A higher dominance of hydrophobic amino acids can enhance gel strength and product stability, while the presence of hydrophilic amino acids may influence the solubility and water absorption capacity of the gelatin [66].

Additionally, cysteine has an impact on gelatin's chemical and physical characteristics, particularly its ability to attract retain water. The cysteine content and WBC in *C. lucius* fish scale gelatin may positively correlate in this study, as cysteine amino acid content was not found in the scale gelatin of *P. bulu* and *C. carpio*. However, the correlation between cysteine levels and WBC has not been analyzed in this study.

In addition, the authors have not conducted studies on potential modifications or adjustments to the formulation for various applications such as edible films, pharmaceutical capsules, or flavor carriers. Therefore, further studies are needed in the future to explore additional purification steps or the use of cross-linking agents to improve the physical properties and stability of the extracted gelatin, especially for high-value products.

On the other hand, this study did not include an assessment of heavy metal content in the gelatin extracted from the three selected freshwater fish species. Nonetheless, previous research has reported the presence of heavy metals in fish scales from various species [32, 67, 68]. Considering the widespread use of gelatin in food applications, it is crucial to determine whether heavy metal concentrations remain within the permissible limits defined for food-grade gelatin. Therefore, further investigation is warranted to evaluate the heavy metal levels in gelatin derived from these species, in compliance with established food safety regulations. Addressing this aspect would enhance understanding of the safety and quality of gelatin, particularly regarding its potential application in the food and pharmaceutical industries.

### 3.10. Limitations of the Study

This study has several limitations related to the extraction method, analyzed parameters, and obtained data:

1. Gelatin extraction efficiency—Although the acid extraction method utilized in this study effectively isolated gelatin, it may have resulted in the partial loss of certain functional properties. The efficiency of gelatin extraction can be influenced by critical factors such as pH, temperature, and reaction time, which were not fully optimized in this study. This lack of optimization may have affected both the consistency and quality of the extracted gelatin. Future research should focus on refining these extraction parameters to improve yield consistency and better preserve the functional properties of gelatin.
2. Amino acid analysis—The amino acid composition of the gelatin reported in this study was obtained from single measurements without replication (*n* = 1) for each of the three selected fish species. The absence of triplicate measurements increases the risk of random variation and potential measurement errors, potentially compromising the accuracy and reliability of the results. To enhance data validity and reduce variability, future studies should incorporate adequate replication.
3. Heavy metal content analysis—This study did not assess the heavy metal content in the extracted gelatin from the selected fish species. This omission represents a significant limitation, as the presence of heavy metals can adversely impact the quality, safety, and potential industrial applications of gelatin, including its use in food, cosmetics, pharmaceuticals, and aquaculture feed formulations. Future studies should address this gap by including comprehensive heavy metal analyses to ensure the safety and regulatory compliance of the produced gelatin for various commercial applications.

## 4. Conclusion

Biometric indicators of three freshwater fish species with different trophic levels showed CFs greater than 1, indicating good health, optimal feed quality, and good environment. All species showed a strong LWR, with *R*^2^ values exceeding 0.77, indicating a significant correlation between body length and weight. The physicochemical and functional properties of gelatin derived from the scales of *P. bulu*, *C. carpio*, and *C. lucius* were influenced by differences in protein composition and specific dietary factors of each species. Gelatin from *P. bulu* showed the highest yield and better FA and FS compared to *C. lucius* and *C. carpio*. In contrast, *C. lucius* gelatin had higher water retention capacity and emulsifying ability and showed the highest gel strength and viscosity, which were most likely due to the higher contents of glycine, proline, and alanine compared to the other two species. The biochemical composition of gelatin also showed variations in water, protein, fat, and ash content, with the highest protein content in *P. bulu*. These findings confirm that the functional properties of gelatin are greatly influenced by amino acid composition and species-specific biological factors. Therefore, further studies are needed to explore the factors that influence the variation of gelatin properties, especially those related to specific amino acid composition, and to evaluate its potential applications in the food, pharmaceutical, biomedical, fish feed formulation, and aquatic animal disease treatment industries to determine its commercial viability.

## Figures and Tables

**Table 1:** Biometric parameter details of three selected freshwater fish species.

**Trophic levels**	**Fish species**	**Results**							
		**Wet weight (g)**	**Standard length (cm)**	**Height (cm)**	**Condition factor**	**b**	**R** ^2^ ** of LWR**	**Growth pattern**	**Scale size (mm)**
Herbivore	*Puntioplites bulu*	207 ± 134.61	18.22 ± 3.04	8.28 ± 2.29	2.97 ± 0.33	3.3206	0.98	(+)	15.99 ± 1.69
Omnivore	*Cyprinus carpio*	597.72 ± 109.19	25.98 ± 1.59	9.61 ± 0.40	3.30 ± 0.10	2.6838	0.77	(−)	15.04 ± 1.88
Carnivore	*Channa lucius*	237.01 ± 83.16	27.33 ± 3.16	6.37 ± 0.73	1.13 ± 0.15	2.7490	0.85	(−)	10.54 ± 0.43

*Note: n* = 25 fish; *n* = 25 scale; (+) = positive allometric; (−) = negative allometric.

Abbreviation: LWR = length–weight relationship.

**Table 2:** Gelatin yield from three selected freshwater fish species. Mean ± SD^*^.

**Trophic levels**	**Species**	**Yield (%)**		
		**Wet basis**	**Dry basis**	**Protein basis**
Herbivore	*Puntioplites bulu*	12.35 ± 0.68^a^	30.50 ± 2.34^a^	42.35 ± 3.39^a^
Omnivore	*Cyprinus carpio*	7.13 ± 0.26^b^	20.98 ± 0.33^b^	30.41 ± 0.41^b^
Carnivore	*Channa lucius*	5.03 ± 0.15^c^	18.02 ± 0.20^c^	26.11 ± 0.29^c^

*Note:* Description: Values are presented as the mean ± standard deviation (percent) based on triplicate measurements. Lowercase superscript letters denote significant variations in three fish species (*p* < 0.05).

^*^The values represent the mean of triplicate samples.

**Table 3:** Amino acid composition (amino acids g/100 g protein) of the three samples.

**Type of amino acids**	**Amino acids**	**Results**		
		***Puntioplites bulu***	***Cyprinus carpio***	***Channa lucius***
Hydrophobic amino acids	Leu	2.70	1.85	1.84
	Val	1.71	1.63	1.53
	Ile	1.35	0.76	0.91
	Trp	0.67	0.34	0.51
	Phe	1.15	1.55	1.58
	Met	1.36	0.51	1.24
	Pro	9.52	9.56	9.78
	Ala	7.93	7.70	8.47
	Gly	18.72	18.69	20.83

Total hydrophobic amino acids		45.11	42.65	46.68

Hydrophilic amino acids	Arg	11.94	11.87	8.29
	His	5.20	4.35	1.46
	Lys	3.59	3.62	3.37
	Thr	2.12	2.16	2.04
	Tyr	0.50	0.23	0.36
	Glu	7.74	7.78	8.56
	Cys	ND	ND	0.15
	Asp	4.53	4.48	4.16
	Ser	2.98	2.88	3.28

Total hydrophilic amino acids		38.61	37.38	31.69

Abbreviation: ND = not detected.

**Table 4:** Mineral composition of gelatin derived from the scales of three samples.

	**Mineral composition**	**Results (mg/kg)**		
		***Puntioplites bulu* scale gelatin powder**	***Cyprinus carpio* scale gelatin powder**	***Channa lucius* scale gelatin powder**
Macromineral (mg/kg)	Sodium (Na)	67.72	102.85	4638.30
	Magnesium (Mg)	532.46	12.26	692.34
	Calcium (Ca)	431.66	6.67	503.45
	Potassium (K)	56.37	68.98	310.46
	Phosphorous (P)	0.35	0.26	0.64

Microminerals (mg/kg)	Zinc (Zn)	3.02	4.91	21.03
	Iron (Fe)	160.58	30.09	72.43
	Manganese (Mn)	0.58	0.64	2.46

## Data Availability

The data that support the findings of this study are openly available in figshare at https://figshare.com/articles/dataset/_b_Biometric_Indicators_Gelatin_Yield_and_Functional_Properties_of_Fish_Scale_Waste_from_Three_Species_with_Different_Trophic_Levels_b_/27967104, Reference Number 10.6084/m9.figshare.27967104.v4.
